# Complex life histories discovered in a critically endangered fish

**DOI:** 10.1038/s41598-019-52273-8

**Published:** 2019-11-14

**Authors:** James A. Hobbs, Levi S. Lewis, Malte Willmes, Christian Denney, Eva Bush

**Affiliations:** 1 Wildlife, Fish and Conservation Biology, University of California, Davis, 1 Shields Ave, Davis, CA 95616 USA; 2 Present Address: Delta Science Program, Delta Stewardship Council, Sacramento, CA USA

**Keywords:** Animal migration, Biodiversity

## Abstract

Effective conservation of endangered species requires knowledge of the full range of life-history strategies used to maximize population resilience within a stochastic and ever-changing environment. California’s endemic Delta Smelt (*Hypomesus transpacificus*) is rapidly approaching extinction in the San Francisco Estuary, placing it in the crossfire between human and environmental uses of limited freshwater resources. Though managed as a semi-anadromous species, recent studies have challenged this lifecycle model for Delta Smelt, suggesting the species is an estuarine resident with several localized “hot-spots” of abundance. Using laser-ablation otolith strontium isotope microchemistry, we discovered three distinct life-history phenotypes including freshwater resident (FWR), brackish-water resident (BWR), and semi-anadromous (SA) fish. We further refined life-history phenotypes using an unsupervised algorithm and hierarchical clustering and found that in the last resilient year-class, the FWR (12%) and BWR (7%) comprised a small portion of the population, while the majority of fish were SA (81%). Furthermore, the semi-anadromous fish could be clustered into at least four additional life-history phenotypes that varied by natal origin, dispersal age and adult salinity history. These diverse life-history strategies should be incorporated into future conservation and management efforts aimed at preventing the extinction of Delta Smelt in the wild.

## Introduction

Species have evolved complex life history strategies to utilize spatially and temporally variable environments^1^. In fishes, diadromy, or the ability to migrate between freshwater and saltwater for the purpose of reproduction, is a common strategy employed to minimize predation risk on small vulnerable young in one habitat, while maximizing growth and reproductive output in another, thereby maximizing lifetime fitness^2,3^. In temperate climates the most common form is anadromy, where adults live in the ocean for several years prior to returning to freshwater for reproduction (e.g. salmonids)^3,4^. However, some anadromous species stop short of migrating to the sea and rear in brackish estuarine habitats^5,6^. Regardless of the direction of movement, the migratory period is a critical part of the lifecycle in diadromous species where substantial risks from both natural (e.g. predation, starvation) and anthropogenic factors (e.g. fishing, entrainment into water diversions) can occur, creating unique challenges for managing and protecting these species.

To spread the risk of catastrophic mortality among habitats, many anadromous species exhibit diverse life history pathways whereby some individuals choose to remain resident in fresh water while others migrate to the sea, such as Steelhead Trout (*Oncorhynchus mykiss*)^7–11^. Similarly, individuals can exhibit considerable variation in outmigration timing, time-at-sea, return timing and size and age at maturity^12–14^. Indeed, this diversity of intra and inter-population life histories can provide populations and species with a degree of stability during periods of poor environmental conditions and resilience following returns to periods of more favorable environmental conditions^15–18^. However, for declining or endangered species, such life-history diversity may be lacking, limiting their ability to respond to improved conditions or management actions employed to recover the species. This may be particularly important when such life-history diversity is cryptic and conservation strategies are aimed to benefit only one life-history strategy.

The Delta Smelt (*Hypomesus transpacificus*) is one of the most socio-politically important species in the Western United States and is nearing extinction in the wild^19–22^. This small, 2–3 inch cucumber-scented fish is endemic to the upper San Francisco Bay-Delta Estuary (SFE), where spawning habitat coincides with the State Water Project (SWP) and Central Valley Project (CVP) pumping facilities which provide fresh water for 23-million people and irrigation for a multi-billion-dollar agriculture industry^21^. Despite numerous protections, conservation efforts, and ecological studies over the past three decades, the population has continued to decline and is now at less than 1% of its historic abundance^22,23^. At times, large numbers of Delta Smelt become entrained in the CVP pumping facilities and other water diversions and killed. This can occur during their upstream spawning migration into fresh water in the late winter and in spring when the larvae reach a size which can be counted in the fish collection facilities at SWP and CVP^23–25^. To protect the species from excessive loss, pumping is restricted from January to June and can be shut down for periods of time when large numbers of fish are entrained, leading to delays or shortages in water deliveries^24^. In addition, freshwater flows into the estuary are required to maintain suitable low-salinity nursery habitat downstream of the agricultural diversions during the late summer and fall months. Collectively, these protections reduce the amount of water available for urban and agricultural use, causing significant political conflict and controversy regarding the efficacy of such expensive actions^19,21^.

**Figure 1:**
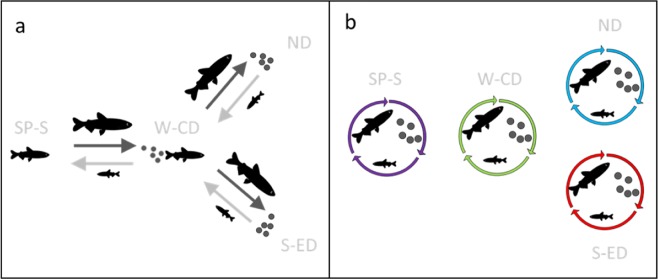
(**a**) The semi-anadromous life-history model for Delta Smelt emphasizing eastward and upstream migration of adults to freshwater spawning areas (winter-spring) followed by downstream and westward movements of larvae and juveniles into brackish rearing habitats^26,31,62^. (**b**) The “hot spot” model indicating local spawning and rearing in fresh and brackish waters^29,52^. SP-S: San Pablo Bay-Suisun Bay; W-CD: West Delta-Central Delta; ND: North Delta; S-ED: South Delta-East Delta.

Delta Smelt has been described as a semi-anadromous species (Fig. 1a); migrating from brackish low-salinity (1–6 PSU) habitats downstream of the confluence of the Sacramento and San Joaquin Rivers to tidal freshwater habitats in the Delta^26^. The spawning migration generally occurs in the winter, following the first flush of turbid freshwater from precipitation events^27,28^. This first-flush period also coincides with increased freshwater availability and elevated pumping rates to fill storage reservoirs for the dry season, thus migration can lead to high adult entrainment and mortality in the South Delta facilities via directed movements^28^. However, recent analyses of long-term monitoring data suggest Delta Smelt do not migrate, rather the species exhibits a resident life history consisting of localized “hot spots” of abundance in brackish and fresh water (Fig. 1b)^29–32^.

Determining the full range of Delta Smelt life-history strategies is critical for devising effective management and conservation strategies for the species. Unfortunately, the species is too small and delicate for traditional tagging methods^33^. However, otoliths (“ear stones”) can record the chemical composition of the ambient water and have been used extensively in estuarine fishes for tracking movements^34–38^. Otoliths have several features that are useful for life history reconstructions; (1) they accrete continuously through life resulting in daily age increments (in the early-life) of calcium carbonate and protein matrix, (2) incorporate elements of the surrounding environment into the calcium carbonate and protein matrix, (3) are biologically inert, (4) and grow in proportion to fish growth allowing for size reconstructions producing a life-long record of growth and environmental conditions. Strontium is an abundant trace element readily substituted for calcium in the mineral lattice, resulting in Sr concentrations and ^87^Sr/^86^Sr values that reflect the ambient water chemistry^39–41^. The ratio of radiogenic ^87^Sr to the stable ^86^Sr in rivers and lakes has proven to be a powerful tracer of provenance in many species because it varies predictably between different watersheds as a function of the age and geochemical composition of the underlying geology^42–45^. In the SFE, ^87^Sr/^86^Sr reflects the conservative mixing of fresh water from the Sacramento and San Joaquin rivers with the Pacific Ocean and has been used to reconstruct the salinity history of individual fish^37,38,46^. Moreover, ^87^Sr/^86^Sr has been shown to provide high resolution for reconstructing rearing from freshwater to low-salinity (<6 PSU) habitats in the upper SFE, where greater than 90% of all Delta Smelt occur.

**Figure 2:**
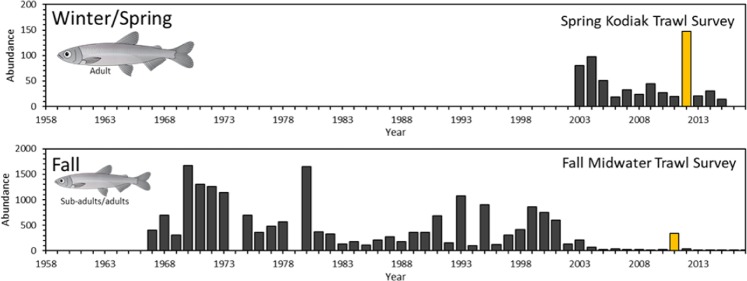
Time series of the population abundance index of Delta Smelt based on the Fall Midwater Trawl Survey and Spring Kodiak Trawl Survey (CDFW). Illustrations of Delta Smelt by Adi Khen.

To resolve the controversy regarding the life-history of Delta Smelt, we combined otolith microstructure measurements (daily increments) with profiles of otolith ^87^Sr/^86^Sr from the core (hatch) to the edge (catch) to reconstruct age-resolved ^87^Sr/^86^Sr chronologies. This analytical approach can provide detailed high-resolution time series of individual life histories but can also lead to computational challenges when evaluating large numbers of individuals. Furthermore, prior studies have primarily relied on professional judgement or qualitative assessment of ^87^Sr/^86^Sr profiles, which can be challenging when attempting to characterize life-history patterns among a large number of individuals. Therefore, we employed an unsupervised time-series algorithm, the Discrete Wavelet Transform, that can efficiently decompose complex time series into shorter, simpler segments while retaining the important time-dependent changes in chronologies^47^. The simplified chronologies were then used in hierarchical agglomerative clustering to identify groups of individuals with similar ^87^Sr/^86^Sr chronologies. The objective of the present study was to describe life-history patterns and their key attributes among a large number of individuals collected during a year when population abundance rebounded from prior low abundance observed during drought (Fig. 2). The application of these tools to quantify life-history diversity in a resilient year class may elucidate mechanisms of recruitment that can inform and enhance water management and conservation strategies aimed at promoting population resilience and persistence of this endangered species.

## Results

### Supervised life-history assignments

**Figure 3:**
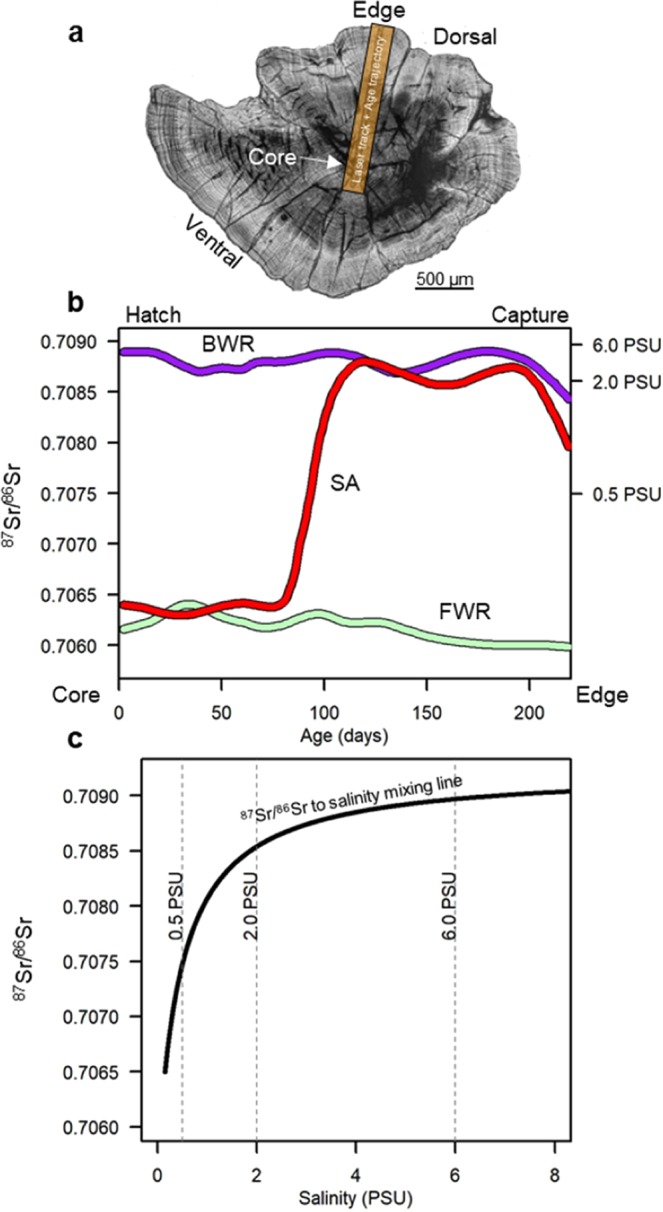
(**a**) Delta Smelt otolith in sagittal plane. (**b**) Three example ^87^Sr/^86^Sr profiles from the core (hatch) to the dorsal edge (capture). (**c**) Simplified ^87^Sr/^86^Sr to salinity mixing model for the San Francisco Estuary. Details and validation of the mixing model is provided in the supplement.

We examined otolith ^87^Sr/^86^Sr profiles of Delta Smelt caught between September 2011 and May 2012 by monitoring surveys, in what was the last year of high abundance for this species up to date (Fig. 2). Three distinct patterns were apparent based on visual inspections of ^87^Sr/^86^Sr profiles (Fig. 3). One group had relatively flat ^87^Sr/^86^Sr profiles where ^87^Sr/^86^Sr values were always below 0.7072, indicative of fish rearing in fresh water based on our knowledge of water ^87^Sr/^86^Sr and salinity reconstructions in the SFE, where the boundary between freshwater (<0.5 PSU) and brackish water (>0.5 PSU) is at ~0.7075 (Fig. 3). A second group had ^87^Sr/^86^Sr profiles that remained above ^87^Sr/^86^Sr values of 0.7075 from core to edge, suggestive of hatching and rearing in brackish water, while a third group had ^87^Sr/^86^Sr values less than 0.7075 during the early life before increasing to above 0.7075, a pattern consistent with semi-anadromy (Fig. 3).

### Unsupervised life history assignments

**Figure 4:**
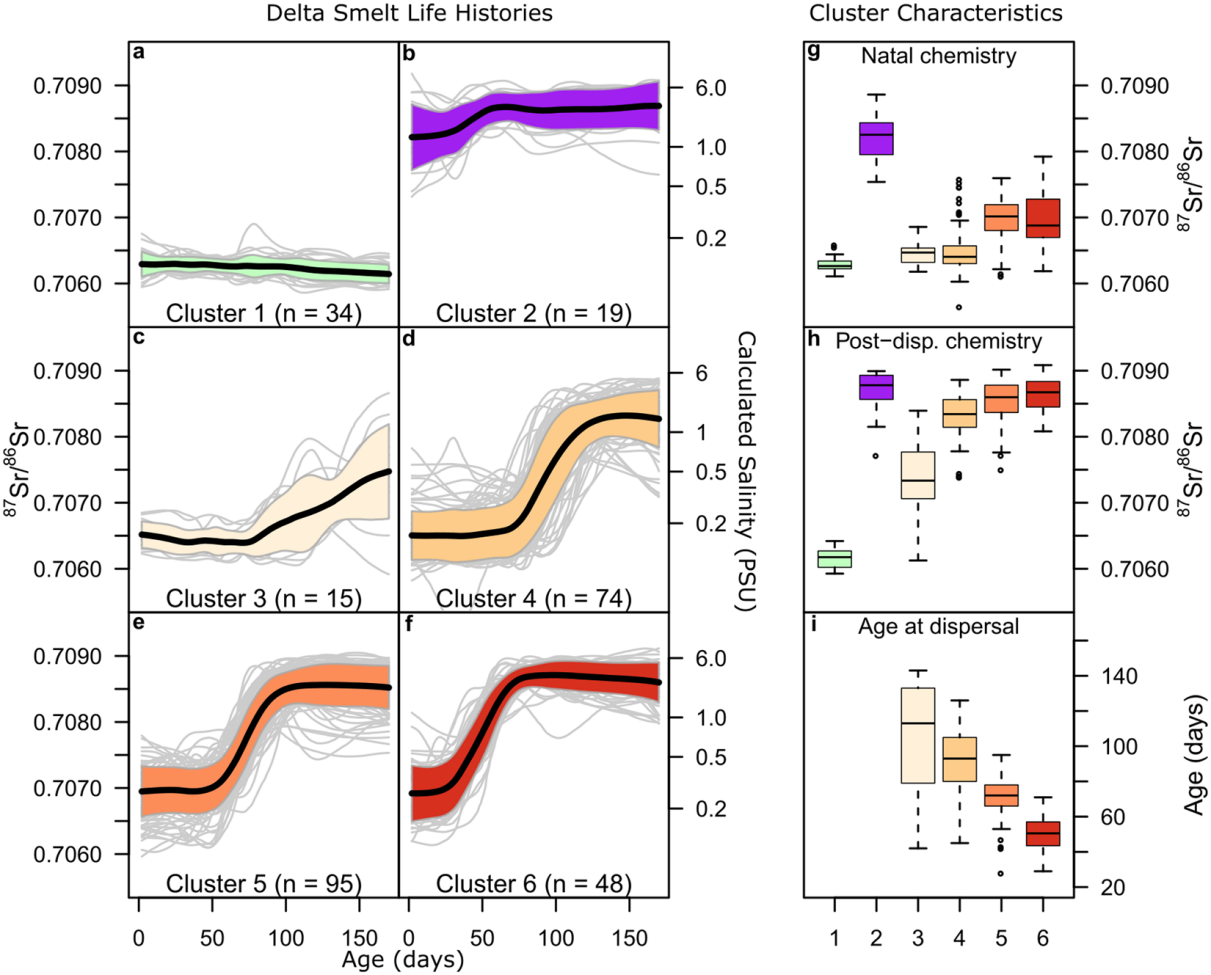
Hierarchical cluster analysis of 285 fish into six clusters based on natal chemistry, post-dispersal chemistry, and age at transition. The mean ^87^Sr/^86^Sr profiles (solid black lines) and ±1σ (shaded area), as well as all individual profiles (light grey lines) from age 0–170 days are shown for all clusters in panels (a–f). Box plots for each cluster are shown for natal chemistry, post dispersal chemistry, and age at transition in panels (g–i).

From the fish analyzed using the supervised approach, a selection process involving consensus age matching and profile length trimming resulted in 285 fish suitable for hierarchical cluster analysis, which created 6 clusters based on the minimization of within cluster variance. The first cluster (Fig. 4a, n = 34) included all fish characterized as freshwater fish while the second cluster (Fig. 4b, n = 19) included all fish that hatched and reared in brackish water. In addition, there were four clusters (Fig. 4c–f, n = 232), containing individuals exhibiting a semi-anadromous pattern, hatching in fresh water and rearing in brackish water to adulthood. Overall, based on the hierarchical clustering, 81% of the fish were characterized as semi-anadromous, 12% freshwater resident and 7% as brackish-water resident.

The clusters could be distinguished based on several life history attributes. Natal origins varied between the clusters, where cluster 1, the freshwater resident fish (Fig. 4a), had the lowest natal ^87^Sr/^86^Sr followed by semi-anadromous clusters 3–4 (Fig. 4c,d), while the semi-anadromous clusters 5–6 (Fig. 4e,f) had elevated natal ^87^Sr/^86^Sr. All of these clusters have natal ^87^Sr/^86^Sr values indicating freshwater natal origins, while cluster 2 had natal ^87^Sr/^86^Sr values suggesting hatching in brackish water (Fig. 4b). The semi-anadromous clusters also varied by their ^87^Sr/^86^Sr values post-dispersal into brackish water and age-at-dispersal from freshwater to brackish water (Fig. 4g–i). Dispersal age for cluster 3 and cluster 4 were on average 113 and 93 days, respectively, and both showed a large range of dispersal ages. Cluster 5 dispersed at around 72 days of age and was different from cluster 6, which dispersed at around 51 days of age, and both showed a smaller range of dispersal ages than cluster 3 and 4. Combining these three key life-history attributes, the four semi-anadromous clusters were significantly different (PERMANOVA p < 0.01) from each other, and could be used to reliably assigned fish to their respective groups using QDFA with an overall Jackknife reclassification success of 90% (Supplement Table S11). When comparing all six clusters, age-at-dispersal was not defined for the two resident groups (FWR, BWR), so we used only natal and post-dispersal chemistry attributes in QDFA, and successful classification decreased to 69% (Supplement Table S12). This was mostly due to the failure to correctly split cluster 5 and cluster 6, indicating that dispersal age is an important feature to differentiate the semi-anadromous groups. When all the semi-anadromous clusters (3–6) were combined, classification success in distinguishing clusters 1, 2, and 3–6 increased to 99% (Supplement Table S13).

### Residency and semi-anadromy

**Figure 5:**
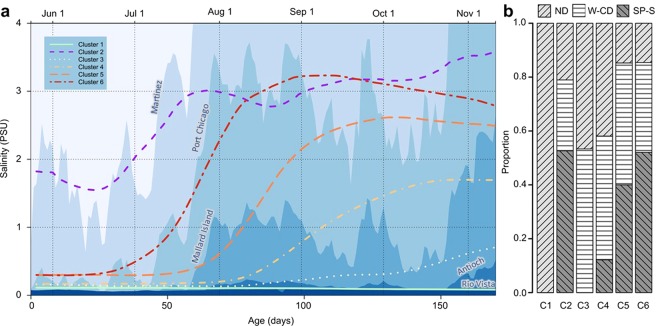
(**a**) Mean salinity profiles of the six Delta Smelt life history strategies (colored lines) overlaid atop continuous salinity profiles (blue contours) measured by water quality measurement sondes at 5 stations throughout the SFE in decreasing mean salinity (Martinez~ San Pablo-Suisun, Port Chicago, Mallard Island, Antioch~ West-Central Delta, Rio Vista~ North Delta). (**b**) Relative abundance of each cluster in distinct regions of the SFE including the San Pablo-Suisun Bays (SP-S); West-Central Delta (W-CD), and North Delta (ND). No fish were caught in the South-East Delta (S-ED) over this period.

Seasonal patterns of *in-situ* salinity profiles at 5 water quality monitoring stations located from the Carquinez Straits to the Delta confluence generally resembled otolith chronologies (Fig. 5). The brackish resident (cluster 2) mean salinity values derived from the ^87^Sr/^86^Sr-salinity mixing curve (Supplement Fig. S2) was consistent with a natal origin near Martinez and the increase in mean salinity was consistent with salinity intrusion at Martinez in early July. However, by the end of July, mean salinity more closely resembled environmental salinity as measured at Port Chicago (Fig. 5). The semi-anadromous clusters 5 and 6 had natal salinity consistent with an origin near Port Chicago and the increase in mean salinity for cluster 6 was consistent with salinity intrusion near this station while the increase in mean salinity in cluster 5 occurred when salinity intrusion reached Mallard Island, suggesting these groups of fish were likely hatched downstream in Suisun Bay and exhibited a resident behavior through the fall (Fig. 5). Mean salinity chronologies in the early life for semi-anadromous clusters 3 and 4 were consistent with natal origins as far downstream as Port Chicago and Mallard Island, but the increase in mean salinity for these clusters occurred later, when salinity intruded beyond these locations. This indicates their natal origins were located upstream and these fish dispersed from freshwater natal habitats into brackish water independent of salinity intrusion.

### Full lifecycles and reproductive strategies

To describe the full life history of the fish, we combined cluster assignments with capture region and gonad maturity stage during the spawning migration season (Jan-May). All freshwater resident fish (cluster 1) were captured in the perennial freshwaters of the northern Delta signifying individuals within this cluster completed their lifecycle in freshwater (Supplemental Table S14). Of the 29 freshwater resident fish, 5 males and 5 females had gonads in a late developmental stage or were in post-spawn condition (reproductively mature) (Supplemental Table S15). Brackish-water resident fish (cluster 2) were caught in all three regions, signifying these fish do migrate to freshwaters during the spawning season (Supplemental Table S14). Furthermore, at least one reproductively mature brackish water resident fish was caught in each region. The largest number of reproductively mature individuals from this group were caught in the North Delta (Supplemental Table S15). Over half (55%) of all semi-anadromous fish (clusters 3–6) were caught in the West-Central Delta and North Delta (Supplemental Table S14). Semi-anadromous male and female fish were mature in all regions (Supplemental Table S15); however, the proportion of mature semi-anadromous fish was greater in the West-Central Delta and North Delta than in San Pablo-Suisun Bay and this appeared to be driven by males (Supplemental Table S15).

**Figure 6:**
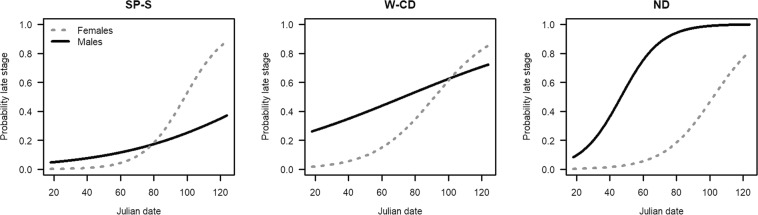
Probabilities of captured fish in early (I–III) vs. late (IV–VI) reproductive stage by Julian date in the three regions split by males and females. SP-S is San Pablo-Suisun Bay, W-CD is Western-Central Delta, and ND is North Delta.

Reproductive maturity for all fish caught in the 2012 Spring Kodiak Trawl Survey increased between January and May (n = 1063) and varied by sex and capture region (Fig. 6). We modeled these changes using a binomial logistic regression including sex, capture region, and Julian date. The full logistic model with all three main effects and all interactions explained 44% of the variability in the response (R^2^_McFadden_ = 0.438),  had the lowest AICc of all examined models (Supplement Table S16), and was significantly different from the null model (p << 0.01, Supplement Table S17) and the next most explanatory model (p << 0.01, Supplement Table S18). The probability of captured fish having late stage gonads increased at approximately day 60 (March) in all regions for females and exceeded 0.75 in all three regions. In males, the shape of the probability curve differed by region. San Pablo-Suisun region had a much lower probability of having late staged gonads than in the West-Central Delta Region and the North Delta (Fig. 6).

## Discussion

Life-history diversity among populations within a species can provide resilience to environmental perturbation and stabilize long-term population trajectories. Protecting such biodiversity may be critical for recovering endangered species in an era of climate change^16,17^. In this study, we discovered life-history diversity within the endangered Delta Smelt in a year-class of high abundance following a drought period. We found the majority of individuals examined were semi-anadromous as originally described; however, a small proportion exhibited a resident life history, residing in fresh or brackish water to adulthood. Interestingly, we also found sexually mature brackish origin fish well upstream in the tidal freshwaters of the northern Delta and semi-anadromous fish remaining downstream in brackish water during the spawning season. This indicates the species spawns in fresh and brackish waters, potentially spreading the risk of catastrophic mortality across habitats. Thus, Delta Smelt exhibited a much more complex life history than previously described, which may provide insights into how such life-history diversity enables population resilience for this endangered fish.

This species evolved from the marine congener *Hypomesus pretiosus* in isolated freshwater lakes formed by receding sea levels during the early Pleistocene^48^, and the complex life histories we observed may represent adaptation to a dynamic mosaic of tidal fresh and brackish habitats found in the historic Delta^49,50^. The distribution and abundance of Delta Smelt has been monitored in the upper SFE as far back as 1959 during summer surveys and 1967 by fall and winter surveys^51^. These long-term monitoring studies have documented the persistence of Delta Smelt in the tidal freshwaters of the lower Sacramento River near the town of Rio Vista year-round, but due to their close proximity to brackish water, it was not acknowledged that Delta Smelt could complete their life cycle in freshwater in the wild^26^. Since 2002, additional surveys conducted in the North Delta have documented year-round presence and, at times, high abundance of Delta Smelt, suggesting freshwater resident fish may be important to the population^31,32,52,53^. Our otolith ^87^Sr/^86^Sr chronologies confirmed the existence of a freshwater resident life history comprising 12% of the adult population in this abundant year-class. With advancements in otolith microchemistry techniques, many anadromous and estuarine species have been confirmed to consist of both freshwater resident and migratory “contingents” within a population, a life-history strategy referred to as partial migration^8,15,54,55^. Moreover, partial migration has been found to provide population resilience during periods of poor estuarine conditions while also promoting long-term population stability by having a portion of the population reside in freshwater^56^. However, when freshwater habitats are highly degraded, as is the case in this estuary, this strategy may lead to poor survival of resident fish and erosion of resilience.

Historically, the North Delta consisted of narrow turbid backwater sloughs bordered by broad sloping natural levees that supported dense riparian forests^49^. Now, the North Delta landscape is dominated by the man-made Sacramento Deepwater Ship Channel and Liberty Island, a large shallow tidal freshwater lake formed accidentally by levee failure in 1998. These habitat features have caused the North Delta to become a backwater area with long hydrodynamic residence time where tidally-averaged flows are commonly landward in the summer and fall and frequently reach temperatures lethal to Delta Smelt^57,58^. The majority of sexually mature fish were found in the North Delta in this study year supporting observations that the North Delta is the primary spawning area for Delta Smelt^23^. Two of our semi-anadromous clusters had natal ^87^Sr/^86^Sr consistent with hatching in the North Delta and their age-at-dispersal into brackish water was much older, suggesting fish hatched in this region may have found it difficult to disperse downstream into the estuary. Recently, studies have found evidence of degraded health in Delta Smelt found in the North Delta during summer months, exhibiting poor feeding, somatic condition and growth rates in addition to histological lesions consistent with contaminant exposure^53,59–61^. Collectively, these studies suggest survival may be impaired for fish in the North Delta and conditions in this region of the estuary may play a large role in the decline of Delta Smelt.

The central and southern Delta are also highly modified habitats, consisting of farmland bordered by rip-rapped levees and channelized sloughs overrun by invasive aquatic weeds and dominated by invasive fishes that compete with or consume Delta Smelt^49,62,63^. The state and federal water projects export freshwater from this region, entraining the vast majority of freshwater entering from the San Joaquin River and a significant fraction of Sacramento River water, causing strong reverse flows in the channels leading to the pumping facilities and resulting in large numbers of adult and larval Delta Smelt being entrained and killed^28,64^. Furthermore, physiochemical habitat attributes have changed in the Delta, turbidity has drastically declined^65^ and summer water temperatures have increased to thermal limits for the Delta Smelt precluding residency in the Central and South Delta in the summer^66^. Taken together, habitat alteration, invasive species and water diversion have made the freshwater regions of the central and southern Delta largely unsuitable for Delta Smelt and the freshwater resident life history.

The Delta Smelt’s semi-anadromous life-history strategy was originally described as an adaptation to take advantage of high zooplankton prey abundance in highly turbid brackish waters (1–6 PSU) found in Suisun Bay and Suisun Marsh, commonly referred to as the Low-Salinity Zone (LSZ)^26^. The LSZ was historically a region of high productivity, but these conditions have drastically changed following the invasion of the overbite clam (*Potamocorbula amurensis*) in 1986, which dramatically reduced phytoplankton and zooplankton abundance^67–69^. Furthermore, capture of sediments by dams^70^ and invasive aquatic vegetation^65^, and reduced resuspension due to decreasing wind velocities^71^ have reduced turbidity throughout much of the Delta, resulting in clearer waters that are less suitable for Delta Smelt. suitability of the LSZ has declined due to reduced sediment supply to the estuary^70^, increased capture by invasive submerged aquatic vegetation^65^ and decreasing wind patterns^71^. Preferred zooplankton prey are now more abundant in freshwater year-round^72^, suggesting fish rearing in the LSZ may be at a disadvantage; however, Hammock, *et al*.^73^ found that Delta Smelt feeding success was higher in brackish habitats in the fall and winter, suggesting semi-anadromous fish were capable of foraging successfully despite lower prey abundance. Furthermore,  they found growth rates for Delta Smelt in the brackish habitats of Suisun Bay and Suisun Marsh were similar or higher than other regions in some years. Thus semi-anadromous fish continue to utilize the LSZ as evident by their larger contribution to the population^53,59^.

Our otolith ^87^Sr/^86^Sr data during the natal period suggest Delta Smelt can utilize brackish habitats successfully for larval rearing; however, the vast majority of adults in this study reared as larvae in freshwater habitats. Delta Smelt are tolerant of brackish water during the juvenile, sub-adult and adult life stages^74–76^, however, the physiological tolerance of larval Delta Smelt has not been tested in the laboratory. While we may infer natal origin from otolith ^87^Sr/^86^Sr in the otolith core, we cannot be certain our data supports spawning in brackish habitats. Using a 3-D hydrodynamic particle tracking model Kimmerer, *et al*.^77^ showed that planktonic organisms could be dispersed rapidly, 10–25 km per day, thus a newly hatched larvae could be dispersed into brackish water within a few days post-hatch. However, we found approximately 50% of brackish and semi-anadromous clusters remained in brackish water through the spawning season, many of which had late stage gonads (stage IV-VI) at capture according to field-based assessment of gonad stage^78,79^. Furthermore, Kurobe, *et al*.^79^ measured 17β-estradiol from livers of females and found late stage maturity (late stage IV) corresponded with spikes in 17β-estradiol, which suggests fish were ready to spawn. Delta Smelt are capable of seasonal batch-spawning, producing multiple clutches of eggs with a refractory period lasting on the order of 40–50 days^63,80^. Given the patterns in life history and reproductive biology, individuals may spawn in both brackish water and freshwater within a reproductive season further spreading the risk of catastrophic mortality across habitats.

The life-history patterns found in this study provide definitive evidence for the original semi-anadromous designation; however, we also found evidence of resident life histories suggesting that Delta Smelt can also occur as population “hot spots” as described by Murphy and Hamilton^29^. The diversity of life histories observed in the study may provide insights into the species’ ability to rebound during periods of good environmental conditions. The estuary experienced high freshwater inflows in 2011, which resulted in greater volume and overlap of the LSZ with marsh habitats found in Suisun Bay. This increased volume of the LSZ corresponded with two groups of Delta Smelt with natal ^87^Sr/^86^Sr consistent with this very low (0.5–1 PSU) salinity. In dry years the volume of this habitat would be reduced and distributed upstream into the narrow-deep channels of the Sacramento-San Joaquin confluence, which likely results in limited production in this marginal habitat. Furthermore, summer water temperature in 2011 was relatively cool, which may have allowed freshwater resident fish to have greater survival and contribution to the adult population. Combined, the cool temperatures and above normal precipitation likely facilitated a greater contribution from more life histories in 2011 allowing the population abundance to increase. Unfortunately, drought conditions began in 2012 resulted  in low abundances and, despite wet conditions in 2017, the population failed to rebound. The population is now at such low abundance that long-term monitoring surveys are struggling to find the fish in the wild. Thus, while life-history diversity may provide some measure of resilience in this species, the degree of habitat alteration and additional stressors continue to depress the population.

Delta Smelt continue to exhibit life history diversity despite near-complete loss of historic habitat across the species' range. However, this hidden life history diversity has led to significant conflict and controversy over how to best manage and conserve this species^21–24,29,30^. Management for this species has come primarily in the form of freshwater flow and export regulations, however; these contentious actions have had mixed results thus far. Given this new description of Delta Smelt life history, conservation efforts need to take a multi-pronged approach to restore habitats in both freshwater and the brackish areas of the upper estuary. Restoration actions may come in the form of both physical habitat rehabilitation and reconnections with marsh habitats, but also freshwater flow actions to provide greater overlap of low-salinity habitat with dynamic physical habitats. While marsh restoration and flow management are available tools for species management in the estuary, rising temperature is an issue that is increasingly becoming intractable. Ultimately, the conservation and recovery of the Delta Smelt will require substantial changes to the management of the estuary, including major tradeoffs between users of California’s natural resources.

## Materials and Methods

### Ethics statement

A California Endangered Species Act (CESU) Memorandum of Understanding (MOU) was made and entered into by and between James Hobbs of the University of California, Davis (permittee) and the California Department of Fish and Wildlife (CDFW). The purpose of the CESA MOU was to authorize the permittee to obtain and possess Delta Smelt (*Hypomesus transpacificus*) collected by the CDFW Interagency Ecological Program for scientific purposes pursuant to Fish and Game Code (FGC) 2081 (a). The portions of the study relating to Delta Smelt were approved by the Institutional Animal Care and Use Committee, University of California, Davis and followed the methodology in the approved protocol (IACUC Protocol #18175). All samples received were archived and reported in according with the CESA MOU and all methods and procedures used in this study were performed in accordance with the guidelines and regulations established in the IACUC protocol.

### Sample collection and subsampling

**Figure 7:**
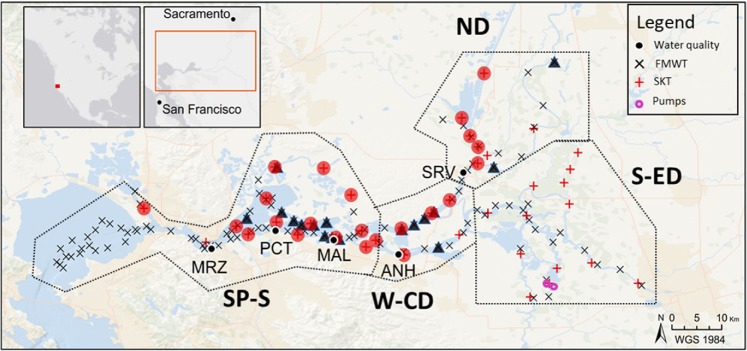
Study region showing sampling stations for the 2011 Fall Midwater Trawl (black x) and 2012 Spring Kodiak Trawl (red+) surveys. Triangles and circles reflect stations from which specimens were collected in the current study. Locations of continuous water quality monitoring stations are indicated by black circles (MRZ-Martinez, PCT - Port Chicago, MAL - Mallard, ANH - Antioch, SRV - Rio Vista). CVP and SWP pumps indicated by magenta circles. The sampling region was divided into 5 regions indicated by dashed lines (San Pablo-Suisun Bays, SP-S; West-Central Delta, W-CD; North Delta, ND; South-East Delta, S-ED).

Delta Smelt were collected from the San Francisco Estuary (SFE) across their entire known geographic range by the California Department of Fish and Wildlife (CDFW) during their 2011 Fall Midwater Trawl (FMWT: Sep-Dec) and 2012 Spring Kodiak Trawl (SKT: Jan-May) surveys (Fig. 7). A total of 326 fish were collected in the FMWT, of which 184 were subsampled. A further 1093 fish were collected in the SKT with 172 subsampled for this study. Subsampling was conducted by stratifying catch by the North Delta and West-Central Delta from San Pablo-Suisun Bay by month, fish length and sex. Fish were selected to maintain proportions caught by each variable. For the unsupervised hierarchical clustering, further selection for age matching and fish age was conducted, resulting in a total of 285 fish, 113 from FMWT and 172 from SKT.

### Otoliths analyses

Sample preparation and analyses was conducted following established protocols ^39,81^. Sagittal otoliths were extracted from the fish, cleaned, and sanded with wet-dry sandpaper until the daily age rings were visible. Otoliths were then imaged at 20X magnification and otolith increments were measured along the dorsal plane approximately 90 degrees from the anterior-posterior axis. Otolith strontium isotope ratios (^87^Sr/^86^Sr) were analyzed at the UC Davis Interdisciplinary Center for Plasma Mass Spectrometry (UCD/ICPMS) using a multi-collector inductively coupled plasma mass spectrometer (*Nu Plasma HR*) interfaced with a Nd:YAG 213 nm laser (New Wave Research UP213). A laser beam of 40 µm diameter traversed across the otolith from ~100 µm before the core to the dorsal edge at 10 µm/s following the same direction as the aging profile (Fig. 3). Data reduction was performed offline using the IsoFishR application^82^ and included a blank subtraction, Rb correction, mass bias normalization and a 2σ outlier criterion using a 40-point moving average window. To merge age increment and ^87^Sr/^86^Sr profiles, we converted the absolute distances to proportional distances and fit a cubic spline (df = 10) to predict the ^87^Sr/^86^Sr onto the age transect, resulting in a daily time series of ^87^Sr/^86^Sr values for each fish.

### Life history assignments

Distinct life history patterns were readily apparent upon visual inspection of ^87^Sr/^86^Sr chronologies (Fig. 3). We used two approaches to assign individuals to life histories. The first, used a supervised procedure to visually assess each profile for distinct changes in the ^87^Sr/^86^Sr using a threshold ^87^Sr/^86^Sr value of 0.7075 to distinguish fresh water from brackish water rearing. This supervised approach was conducted on 430 fish. Fish were classified as freshwater resident (FWR) when entire profiles were less than 0.7075, as semi-anadromous (SA) when the natal period (first 30-days post hatch) was less than 0.7075 and at older life-stage greater than 0.7075, and as brackish-water origin (BWR) when the natal and whole-life profiles were greater than 0.7075. Note that we did not use the otolith edge ^87^Sr/^86^Sr values in this classification scheme as some fish exhibited clear changes in ^87^Sr/^86^Sr values that would be interpreted as a return to freshwater from low-salinity in the late winter, but many samples did not show this despite being caught in freshwater, thus life history assignments are from hatch to the sub-adult phase. To determine complete life-cycle migrations we used capture region and salinity-at-capture along with otolith edge ^87^Sr/^86^Sr values to infer returns to freshwater.

The second approach to assign life history used an unsupervised hierarchical time series clustering algorithm to group individual fish based on their ^87^Sr/^86^Sr chronologies. The daily resolved life history chronologies were decomposed into shorter, simpler chronologies using a Discrete Wavelet Transform (DWT) with a Haar wavelet. The use of wavelets has gained increasing use in non-stationary time series analysis as this approach can efficiently decompose a complex signal on local time-scales, retaining the time-dependent nature of the data while providing simplification of the signal^83^. The DWT has been used successfully in many fields including economics, computer science, and ecology to decompose time series into coefficients that allow for more efficient comparisons of low and high frequency variation^47,84,85^. Wavelet coefficients from the approximation sub-bands at the first level of decomposition were used to calculate Euclidean distance measures between age-matched individuals. Groups of fish were identified using the Euclidean distance measures in an agglomerative hierarchical clustering algorithm using the Ward’s method. We limited the cluster tree to 6 nodes to minimize the number of clusters while capturing an adequate level of life history detail. The DWT and agglomerative hierarchical clustering were performed using the tsClust package in R^47^. One limitation of this approach is that all time series examined need to be the same length for clustering, thus we trimmed otolith chronologies to the first 170 days of age. We chose this length of time because the oldest/latest fish to have moved from freshwater to brackish water based on the supervised approach was 143 days of age. We chose a buffer of 30-days to ensure inclusion of sufficient post-transition signal while also minimizing the number of fish removed from the unsupervised analysis.

To determine when fish migrated, we used changepoint analysis with AMOC (at most one changepoint) on all fish classified as semi-anadromous with the threshold or DWT^86^. The life history cluster and age-at-transition from changepoint analysis was verified by visual inspection of ^87^Sr/^86^Sr chronologies and we found strong agreement between the unsupervised assignments and expert opinion assignments.

We examined variability between DWT clusters using three key life history characteristics: (1) natal origin ^87^Sr/^86^Sr age-0 to age-30, (2) age-at-dispersal from fresh to brackish habitats (inferred from our isotope-salinity mixing models), and (3) post-dispersal ^87^Sr/^86^Sr (age-140 to age-170) reflective of fish living among different salinity zones. We tested group differences using these three life history characteristics with non-parametric permutational MANOVA and assessed classification using Jackknife reclassification success of individuals to their respective life history groups using quadratic discriminant function analysis.

### Evaluation of upstream migration and reproductive stage

To evaluate whether Delta Smelt exhibit semi-anadromous upstream migrations from brackish waters to fresh waters for reproduction, we explored reproductive stage of fish included in our hierarchical clustering along with the capture region and sex. Reproductive state of all adult Delta Smelt collected during the 2012 SKT survey (n = 1,025 fish) was assessed by CDFW and staged as I-immature to VI-spent according the protocols developed in Damon, *et al*.^78^. These stages were further aggregated into early- (I–III) and late- (IV–VI) stage gonads. Since our subsample of fish from this survey included in the hierarchical clustering was small relative to the total number of fish captured representing a description of full life histories, we also modeled the probability of maturity (logistic regression) as a function of Julian date, sex, and region (San Pablo-Suisun, West-Central Delta, North Delta) for all fish caught to explore whether regional differences in reproductive development supported a model of upstream migration for the purposes of reproduction. Of the possible models using these predictors, we used the following model:

$${\rm{Stage}} \sim {\rm{JD}}\,+{\rm{Sex}}+{\rm{Reg}}+{\rm{JD}}:{\rm{Sex}}+{\rm{JD}}:{\rm{Reg}}+{\rm{Sex}}:{\rm{Reg}}+{\rm{JD}}:{\rm{Sex}}:{\rm{Reg}}$$

where Stage is the aggregated reproductive stage, JD is the Julian date, and Reg is the geographical region in which the fish was caught. We included the main effects of Julian date, sex, and region because we think they are important in determining reproductive stage. We included the various interaction terms, because we believe reproductive stage is driven differentially by the interactions of these terms. Models were compared using AICc, R^2^ and χ^2^ tests.

## Supplementary information

Supplementary Information

## Data Availability

The datasets generated during this study are available via GitHub https://github.com/szjhobbs.
