# Supplementary material for: Complex life histories discovered in a critically endangered fish
Source: Sci Rep. 2019 Nov 14;9:16772. doi: 10.1038/s41598-019-52273-8 (PMC6856525; doi:10.1038/s41598-019-52273-8)
Supplement: Supplementary file 1 — Supplementary Information [file 41598_2019_52273_MOESM1_ESM.docx]

**Complex life histories discovered in a critically endangered fish**

Authors: James A. Hobbs*^1^, Levi S. Lewis^1^, Malte Willmes^1^, Christian Denney^1^, Eva Bush^1, 2^

Affiliations:

* corresponding author email: JAHobbs@ucdavis.edu

^1^ Wildlife, Fish and Conservation Biology, University of California, Davis, 1 Shields Ave, Davis CA. 95616, USA.

^2^ Now at Delta Science Program, Delta Stewardship Council, Sacramento, CA, USA

# Supplementary materials

## Study Site

The San Francisco Estuary (SFE) is the largest estuary on the West Coast of the United States of America receiving the majority of its freshwater from the north via the Sacramento River and the south from the San Joaquin River ^1^. These rivers meet to form a tidal delta consisting of a complex network of approximately 1,000 km of interconnected leveed channels protecting islands of farmland and residential areas that were once vast marsh habitats, reclaimed in the early 1900’s. Freshwater inflow to the Delta varies greatly, both seasonally and interannually, reflecting the Mediterranean climate with cool-wet winters and dry-hot summers ^2^. Flows into the Delta are highly regulated by reservoirs with a capacity of about half of the median annual runoff (1906-2010) from the Sacramento–San Joaquin drainage ^3^. In the south Delta, on an annual basis, nearly two-thirds of freshwater inflow is diverted out of the Delta by the State Water Project and the Central Valley Project which sends water to the southern Central Valley and southern California via the California Aqueduct and the Delta-Mendota Canal ^3^. The freshwater inflows to the Delta not diverted (Delta Outflow) move downstream of the confluence of the Sacramento and San Joaquin River to mix with higher salinity water in Suisun Bay. The bulk of Delta Outflow occurs to maintain freshwater conditions (electrical conductivity <0.45 mS) at two points in the Delta (Emmaton and Jersey Point) to meet water quality objectives for the south Delta water projects ^4^.

*Fish Surveys*

Samples for this study were collect by the California Department of Fish and Wildlife’s long-term monitoring surveys of the San Francisco Bay-Delta Estuary. Specifically, we collected fish from two monitoring programs, the Fall Midwater Trawl (FMWT) and the Spring Kodiak Trawl (SKT). The FMWT Survey has sampled the pelagic waters of the upper estuary since 1967 (further details and data can be found at https://www.wildlife.ca.gov/Conservation/Delta/Fall-Midwater-Trawl). The midwater trawl net has mouth dimension of 3.6 width by 3.6-m height, and consists of 9 mesh panels graduating from 20.3-cm at the mouth to 1.3-cm at the cod-end. The survey is conducted monthly from September to December, with each survey consisting of a single 12-minute oblique tow conducted at 100 fixed stations from San Pablo Bay to the Sacramento-San Joaquin Delta. The FMWT Survey captures sub-adult and adult Delta Smelt greater than ~40-mm FL (mean length 60.2-mm FL). The SKT Survey began in 2002 and is conducted monthly from January to May among 40 of the 100 fixed stations sampled by FMWT. The Kodiak trawl net has a mouth opening of 7.6-m width by 1.8-m depth consisting of five panels graduating from 5-cm at the mouth to 0.64-cm at the cod-end. At each station a single 10-minute tow is conducted at the surface. The SKT Survey captures adult Delta Smelt greater than 45-mm FL during sexual maturation (mean length = 65=mm FL), and assesses Delta Smelt gonad stage and sex (further details and data can be found at https://www.wildlife.ca.gov/Conservation/Delta/Spring-Kodiak-Trawl).

*Otolith Sample Selection*

Delta Smelt collected in this survey were sub-sampled for otolith analysis in proportion to the total number of fish captured across the survey regions, survey months, sex (SKT only) and length to maximize inference of life history patterns to the population scale. Catch proportions were similar between the SKT survey and the sub sample used for clustering across month, region, and sex (Tables S1-S4). The FMWT survey diverged significantly in catch proportion from the sub sample (Tables S5-S8). This is largely because most fish caught early in the survey were younger than the cutoff age of 170 days. By the end of the survey (December of 2011), catch proportions and sub-sample proportions were aligned. Similarly, mean length between the fish in the clusters showed no signs of undue divergence (Table S9). The two surveys were subsampled for different reasons. The SKT survey was subsampled because there were too many fish to analyze them all for strontium isotope ratios. Before ^87^Sr/^86^Sr analysis, efforts were made to representatively sample from region and sex. The FMWT survey however had a small number of fish so that all fish were analyzed for strontium isotope ratios. However, most fish caught in the survey, particularly in the early months, were younger than the cutoff age of 170 days.

Table S1: Catch numbers in the SKT survey by month, region, and sex.

| Region | Jan | | Feb | | Mar | | Apr | | May | |
| --- | --- | --- | --- | --- | --- | --- | --- | --- | --- | --- |
|  | male | female | male | female | male | female | male | female | male | female |
| Central Delta | 0 | 0 | 3 | 14 | 0 | 0 | 5 | 3 | 0 | 0 |
| East Delta | 0 | 0 | 0 | 0 | 0 | 0 | 1 | 1 | 0 | 0 |
| Napa River | 0 | 0 | 1 | 1 | 0 | 0 | 0 | 0 | 0 | 0 |
| North Delta | 78 | 40 | 26 | 44 | 44 | 123 | 12 | 27 | 10 | 64 |
| Suisun Bay | 56 | 67 | 61 | 92 | 0 | 0 | 22 | 56 | 4 | 14 |
| West Delta | 21 | 32 | 20 | 10 | 10 | 23 | 6 | 14 | 2 | 21 |

Table S2: Sub sample numbers used in clustering from the SKT survey by month, region and sex.

| Region | Jan | | Feb | | Mar | | Apr | | May | |
| --- | --- | --- | --- | --- | --- | --- | --- | --- | --- | --- |
|  | male | female | male | female | male | female | male | female | male | female |
| Central Delta | 0 | 0 | 0 | 0 | 0 | 0 | 0 | 0 | 0 | 0 |
| East Delta | 0 | 0 | 0 | 0 | 0 | 0 | 0 | 0 | 0 | 0 |
| Napa River | 0 | 0 | 1 | 1 | 0 | 0 | 0 | 0 | 0 | 0 |
| North Delta | 14 | 8 | 2 | 4 | 7 | 15 | 3 | 11 | 4 | 8 |
| Suisun Bay | 9 | 20 | 9 | 5 | 0 | 0 | 3 | 10 | 0 | 0 |
| West Delta | 4 | 7 | 3 | 2 | 5 | 4 | 2 | 2 | 0 | 0 |

Table S3: Catch percentages from the SKT survey by month, region, and sex. Proportions within a given month, including both male and female, add up to 100%.

| Region | Jan | | Feb | | Mar | | Apr | | May | |
| --- | --- | --- | --- | --- | --- | --- | --- | --- | --- | --- |
|  | male | female | male | female | male | female | male | female | male | female |
| Central Delta | 0 | 0 | 1.1 | 5.1 | 0 | 0 | 3.4 | 2 | 0 | 0 |
| East Delta | 0 | 0 | 0 | 0 | 0 | 0 | 0.7 | 0.7 | 0 | 0 |
| Napa River | 0 | 0 | 0.4 | 0.4 | 0 | 0 | 0 | 0 | 0 | 0 |
| North Delta | 26.5 | 13.6 | 9.6 | 16.2 | 22 | 61.5 | 8.2 | 18.4 | 8.7 | 55.7 |
| Suisun Bay | 19 | 22.8 | 22.4 | 33.8 | 0 | 0 | 15 | 38.1 | 3.5 | 12.2 |
| West Delta | 7.1 | 10.9 | 7.4 | 3.7 | 5 | 11.5 | 4.1 | 9.5 | 1.7 | 18.3 |

Table S4: Catch percentages from the SKT subsample used in clustering. Proportions within a given month, including both male and female, add up to 100%.

| Region | Jan | | Feb | | Mar | | Apr | | May | |  |
| --- | --- | --- | --- | --- | --- | --- | --- | --- | --- | --- | --- |
|  | male | female | male | female | male | female | male | female | male | female | |
| Central Delta | 0 | 0 | 0 | 0 | 0 | 0 | 0 | 0 | 0 | 0 | |
| East Delta | 0 | 0 | 0 | 0 | 0 | 0 | 0 | 0 | 0 | 0 | |
| Napa River | 0 | 0 | 3.7 | 3.7 | 0 | 0 | 0 | 0 | 0 | 0 | |
| North Delta | 22.6 | 12.9 | 7.4 | 14.8 | 22.6 | 48.4 | 9.7 | 35.5 | 33.3 | 66.7 | |
| Suisun Bay | 14.5 | 32.3 | 33.3 | 18.5 | 0 | 0 | 9.7 | 32.3 | 0 | 0 | |
| West Delta | 6.5 | 11.3 | 11.1 | 7.4 | 16.1 | 12.9 | 6.5 | 6.5 | 0 | 0 | |

Table S5: Catch numbers from the FMWT survey.

| Region | Sep | Oct | Nov | Dec |
| --- | --- | --- | --- | --- |
| North Delta | 2 | 3 | 17 | 8 |
| Suisun Bay | 33 | 42 | 17 | 34 |
| West Delta | 2 | 7 | 6 | 154 |

Table S6: Catch numbers from the FMWT subsample used in clustering.

| Region | Sep | Oct | Nov | Dec |
| --- | --- | --- | --- | --- |
| North Delta | 0 | 0 | 13 | 4 |
| Suisun Bay | 0 | 1 | 1 | 19 |
| West Delta | 0 | 1 | 5 | 69 |

Table S7: Catch percentages from the FMWT survey. Each month adds up to 100%.

| Region | Sep | Oct | Nov | Dec |
| --- | --- | --- | --- | --- |
| North Delta | 5.4 | 5.8 | 42.5 | 4.1 |
| Suisun Bay | 89.2 | 80.8 | 42.5 | 17.3 |
| West Delta | 5.4 | 13.5 | 15 | 78.6 |

Table S8: Catch percentages from the FMWT subsample used in the clustering. Each month adds up to 100%.

| Region | Sep | Oct | Nov | Dec |
| --- | --- | --- | --- | --- |
| North Delta | 0 | 0 | 68.4 | 4.3 |
| Suisun Bay | 0 | 50 | 5.3 | 20.7 |
| West Delta | 0 | 50 | 26.3 | 75 |

Table S9: Mean and standard deviation in fork length of each cluster, the full catch of each survey, and of the subsample used in the clustering of each survey.

| Group | Mean FL | SD FL |
| --- | --- | --- |
| Cluster 1 | 62.2 | 4.85 |
| Cluster 2 | 61.7 | 5.25 |
| Cluster 3 | 63.7 | 6.14 |
| Cluster 4 | 64.2 | 4.81 |
| Cluster 5 | 63.1 | 4.46 |
| Cluster 6 | 61.1 | 5.6 |
| FMWT | 60.8 | 6.62 |
| SKT | 64.5 | 5.29 |
| FMWT sub | 60.3 | 4.26 |
| SKT sub | 64.56 | 4.80 |

## Otolith processing

Sagittal otoliths were dissected from the heads of Delta Smelt and stored dry in ThermoScientific Cell Culture Plates. Before mounting, the membrane remains surrounding the otoliths were removed by soaking in 95% ethanol for a minimum of 24 hours. Once the membrane was removed, otoliths were mounted onto microscope glass slides with Crystalbond (509) thermoplastic resin in the sagittal plane. Otoliths were sanded sulcus side up until the outermost rings were visible, turned and sanded with wet-dry sandpaper (Buehler 800 and 1200 grit) until the core rings were visible and then polished with a polishing cloth and 0.3µ polishing alumina. Otoliths were digitized with a 12-megapixel digital camera attached to an Olympus CH30 compound microscope at a magnification of 20X, using AM Scope (MU1000). Otolith increments were enumerated and the increment width and radial distance (μm) from the core to each daily ring was measured using Image-J NIH software (v1.51, https://imagej.nih.gov/ij/). In a previous study we validated the daily periodicity of Delta Smelt otolith increments ^5^. Aging transects followed the dorsal plane of the otolith at about 90 degrees from the anterior-posterior axis.

## Otolith strontium isotope analysis

Polished otoliths were mounted on petrographic slides (~20 per slide) for otolith microchemistry. Otolith strontium isotope ratios (^87^Sr/^86^Sr) were analyzed using established protocols at the UC Davis Interdisciplinary Center for Plasma Mass Spectrometry (http://icpms.ucdavis.edu/). A multi-collector inductively coupled plasma mass spectrometer (*Nu Plasma HR* from Nu Instrument Inc.) was interfaced with a Nd:YAG 213nm laser (New Wave Research UP213) for in situ strontium isotopic measurement by laser ablation (LA-MC-ICP-MS). Helium was used as the carrier gas to maximize sensitivity and minimize sample deposition at the ablation site and was mixed with argon gas between the laser sample cell and the plasma source, for better plasma stability. A laser beam of 40 µm diameter traversed across the otolith from ~100 µm before the core to the dorsal edge at 10 µm per second, with the laser pulsing at 10 Hz frequency resulting in 5-10 J/cm^2^ photon output. Digital images of aging transects were used to place laser profiles along the transect used for age increment measurement to facilitate merging age and laser profiles to create an ^87^Sr/^86^Sr chronology.

Processing of otolith chemistry data was performed using the IsoFishR application^6^. Gas blank and background signals were monitored until ^84^Kr and ^86^Kr stabilized after the sample change (i.e. exposing sample cell to the air) and were measured for 30 seconds and subtracted from the raw ratios. Strontium isotope ratios (^87^Sr/^86^Sr) were internally normalized by the measured ^86^Sr/^88^Sr ratio relative to assumed ratio of 0.1194, which corrects for mass discrimination. Rubidium on mass 85 was monitored to account for any ^87^Rb interference on ^87^Sr. We applied a 5-point integration time and a 20-point moving average to the raw data. Outliers were removed based on 2σ outlier criterion using a 40-point moving average window.

Operating conditions and reproducibility of the LA-MC-ICP-MS were evaluated using in-house reference materials consisting of a modern marine coral from the South China Sea and a modern marine otolith from a White Seabass (*Atractoscion nobilis*) collected offshore of Baja California. Replicate analyses for the coral yielded a mean ^87^Sr/^86^Sr value of 0.70921 ± 0.00006 (n=32, ±2σ) and for the otolith of 0.70919 ± 0.00005 (n=59, ±2σ). These values are in good agreement with the global average ^87^Sr/^86^Sr value of modern seawater of ~0.70918 ^7,8^. To minimize error in merging age increment and ^87^Sr/^86^Sr profiles, we transformed age and ^87^Sr/^86^Sr laser profiles from distance to core (µm) to proportional distances. Then we fit a cubic spline (df = 10) to the ^87^Sr/^86^Sr profile and predicted ^87^Sr/^86^Sr onto the age transect, resulting in a time series of ^87^Sr/^86^Sr values.

***Collection and analysis of water for strontium isotopes***

To validate our understanding of the mixing properties of freshwater endmembers entering the Delta we collected water samples over three months in 2012. Water samples and water quality parameters including electrical conductivity (μs/cm), temperature (°C) were collected from surface water grabs at select stations during California Department of Fish and Wildlife’s Spring Kodiak Trawl survey (Supplement Table S10). The water samples were filtered through a 0.45 μm filter (WhatmanTM Puradisk) into a 250 mL polypropylene container and acidified by adding 1 mL of 3% nitric acid. Samples were then transported to a class 100 laboratory at the UC Davis Interdisciplinary Center for Plasma Mass Spectrometry. Element concentrations were measured using an Agilent 7500ce quadrupole inductively coupled plasma mass spectrometer. For strontium isotopic analysis an aliquot of each water sample was made at volume totaling 1 ng of total strontium. These samples were evaporated to dryness and reconstituted in double-distilled nitric acid (8M) for Sr chromatographic separation using micro-column packed with Sr spec resin (Eichrom Inc.). After separation the samples were dried and reconstituted in 2% double-distilled nitric acid and analyzed with the Nu Plasma HR (MC-ICP-MS). Samples were introduced into the mass spectrometer with a desolvating nebulizer system (DSN-100). Replicate analyses of NIST SRM 987 (strontium carbonate) were conducted bracketing every six samples and normalizing for instrument drift between sessions. An in-house modern coral reference material was processed in parallel with each water sample set and resulted in a mean ^87^Sr/^86^Sr ratio of 0.709182±0.000017 (2σ, n=8) showing high precision and accuracy.

Table S10: Water samples collected throughout the San Francisco Estuary in 2012.

| Sample Date | Region | Location | Specific Conductance (μS/cm) | Strontium Concentration (ppm) | ^87^Sr/^86^Sr | Temperature (°C) | Salinity (PSU) |
| --- | --- | --- | --- | --- | --- | --- | --- |
| 5/2/2012 | North Delta | North Delta | 104 | 63 | 0.706535 | 13.8 | 0.0616008 |
| 5/2/2012 | North Delta | North Delta | 117 | 67 | 0.706462 | 17.7 | 0.065426 |
| 4/4/2012 | North Delta | North Delta | 128 | 108 | 0.705926 | 13.3 | 0.0771054 |
| 4/4/2012 | North Delta | North Delta | 130 | 73 | 0.705903 | 17.1 | 0.07337 |
| 4/4/2012 | North Delta | North Delta | 131 | 76 | 0.705799 | 18.3 | 0.0725311 |
| 5/2/2012 | West Delta | Lower Sacramento River | 135 | 77 | 0.706355 | 18.8 | 0.074138 |
| 5/2/2012 | West Delta | Lower Sacramento River | 139 | 77 | 0.706403 | 13.7 | 0.0833584 |
| 5/2/2012 | West Delta | Lower Sacramento River | 140 | 78 | 0.706296 | 10.8 | 0.0889218 |
| 4/4/2012 | West Delta | Lower Sacramento River | 162 | 88 | 0.705846 | 12.5 | 0.1000296 |
| 4/4/2012 | West Delta | Lower Sacramento River | 174 | 95 | 0.705903 | 10.7 | 0.1120242 |
| 3/8/2012 | North Delta | North Delta | 176 | 95 | 0.705758 | 11.1 | 0.1123716 |
| 5/1/2012 | West Delta | Confluence | 185 | 96 | 0.706235 | 13 | 0.113574 |
| 3/8/2012 | West Delta | Lower Sacramento River | 186 | 98 | 0.705935 | 17.4 | 0.1045478 |
| 4/4/2012 | North Delta | North Delta | 186 | 96 | 0.705758 | 11.9 | 0.1169573 |
| 4/3/2012 | West Delta | Confluence | 198 | 95 | 0.706081 | 18.9 | 0.1082146 |
| 5/1/2012 | Bay | Grizzly-Honker Bay | 218 | 107 | 0.706441 | 18.8 | 0.1193572 |
| 5/1/2012 | West Delta | Confluence | 224 | 112 | 0.706379 | 16.9 | 0.1273459 |
| 5/2/2012 | North Delta | North Delta | 229 | 12 | 0.706318 | 14.1 | 0.1381458 |
| 4/3/2012 | West Delta | Confluence | 232 | 103 | 0.706245 | 12.5 | 0.1450951 |
| 3/8/2012 | West Delta | Lower Sacramento River | 233 | 117 | 0.705885 | 10.9 | 0.1512634 |
| 4/3/2012 | West Delta | Lower San Joaquin | 238 | 114 | 0.706335 | 12.9 | 0.1476246 |
| 3/7/2012 | West Delta | Lower Sacramento River | 244 | 125 | 0.706064 | 13 | 0.1511219 |
| 3/7/2012 | West Delta | Lower Sacramento River | 249 | 124 | 0.705959 | 13.4 | 0.1529217 |
| 5/1/2012 | West Delta | Lower San Joaquin | 250 | 122 | 0.707214 | 17 | 0.1420295 |
| 4/4/2012 | North Delta | North Delta | 273 | 147 | 0.706096 | 17.3 | 0.1543118 |
| 3/8/2012 | North Delta | North Delta | 274 | 145 | 0.705981 | 12.3 | 0.1731327 |
| 5/2/2012 | North Delta | North Delta | 290 | 152 | 0.706535 | 12.8 | 0.1814396 |
| 4/4/2012 | North Delta | North Delta | 312 | 182 | 0.706518 | 17.2 | 0.1771028 |
| 4/5/2012 | Bay | Suisun Bay | 314 | 110 | 0.706561 | 12.5 | 0.1983804 |
| 3/8/2012 | North Delta | North Delta | 315 | 133 | 0.705954 | 12.7 | 0.198084 |
| 5/1/2012 | West Delta | Confluence | 316 | 116 | 0.706521 | 12.6 | 0.1992091 |
| 4/3/2012 | Bay | Grizzly-Honker Bay | 318 | 113 | 0.706547 | 17.1 | 0.1809611 |
| 5/3/2012 | Bay | Montezuma Slough | 358 | 117 | 0.706745 | 10.8 | 0.2369692 |
| 3/8/2012 | North Delta | North Delta | 365 | 195 | 0.706354 | 17.9 | 0.2046462 |
| 5/2/2012 | North Delta | North Delta | 381 | 193 | 0.706495 | 18.2 | 0.2123989 |
| 4/5/2012 | Bay | Montezuma Slough | 391 | 120 | 0.706574 | 11 | 0.2583875 |
| 4/4/2012 | North Delta | North Delta | 425 | 221 | 0.706345 | 12.4 | 0.2720071 |
| 3/8/2012 | North Delta | Deepwater Shipping Channel | 554 | 204 | 0.706264 | 13.6 | 0.3474813 |
| 4/4/2012 | North Delta | Deepwater Shipping Channel | 590 | 203 | 0.706267 | 12.9 | 0.3774655 |
| 5/3/2012 | Bay | Suisun Bay | 690 | 151 | 0.707199 | 13.1 | 0.441915 |
| 3/6/2012 | West Delta | Lower San Joaquin | 992 | 206 | 0.707381 | 16.3 | 0.5957457 |
| 4/5/2012 | Bay | Suisun Bay | 1170 | 212 | 0.707904 | 13.2 | 0.7646219 |
| 5/3/2012 | Bay | Grizzly-Honker's Bay | 1301 | 232 | 0.707791 | 12.6 | 0.8678367 |
| 4/5/2012 | Bay | Suisun Bay | 1344 | 215 | 0.707893 | 13.1 | 0.8863884 |
| 5/3/2012 | Bay | Montezuma Slough | 1538 | 268 | 0.707869 | 13.7 | 1.0054599 |
| 5/3/2012 | Bay | Montezuma Slough | 1849 | 312 | 0.707963 | 13.2 | 1.2359722 |
| 4/3/2012 | Bay | Grizzly-Honker Bay | 2327 | 327 | 0.708347 | 18.3 | 1.3876357 |
| 4/5/2012 | Bay | Suisun Bay | 2372 | 339 | 0.708357 | 13.4 | 1.5987933 |
| 5/3/2012 | Bay | Grizzly-Honker Bay | 2802 | 372 | 0.708458 | 18.4 | 1.684205 |
| 5/3/2012 | Bay | Suisun Bay | 3000 | 420 | 0.708449 | 16.5 | 1.8954675 |
| 5/3/2012 | Bay | Suisun Bay | 4263 | 556 | 0.708664 | 16.5 | 2.7518144 |
| 5/3/2012 | Bay | Suisun Bay | 7470 | 952 | 0.708906 | 16.3 | 5.0342937 |
| 4/5/2012 | Bay | Napa River | 11140 | 1447 | 0.708997 | 16.4 | 7.7190359 |
| 5/3/2012 | Bay | Napa River | 16530 | 2126 | 0.709074 | 17 | 11.659146 |

## Estimating salinity based on strontium isotope ratios

Strontium isotope ratios (^87^Sr/^86^Sr) have been used in a variety of provenance studies as a natural tracer of heterogeneous landscapes^9^. Spatial variation in ^87^Sr/^86^Sr values within watersheds is derived from bedrock age and composition as the weathering of different types of rock influence the Sr concentration and ^87^Sr/^86^Sr released into the watershed^10,11^. ^87^Sr/^86^Sr values in biogenic carbonates, such as fish otoliths are largely derived from the surrounding water, with very small influence from diet contributing to otolith ^87^Sr/^86^Sr values ^8^. Thus, otolith ^87^Sr/^86^Sr values can be used to reconstruct origins and migratory history for fishes in freshwater. Since the half-life of ^87^Sr is on the order of 4.5 billion years, and the ocean exhibits a relatively long turnover time for strontium, the modern ocean has a stable ^87^Sr/^86^Sr mean value of ~0.70918^12,13^. When fresh river waters flow into enclosed bays, consistent spatial/longitudinal gradients in salinity occur and the mixing of fresh and ocean ^87^Sr/^86^Sr follows a conservative linear mixing process, such that ^87^Sr/^86^Sr values can be used to estimate salinity with relatively high precision^14-16^. This mixture is largely dependent on the bulk concentration of Sr, salinity, and ^87^Sr/^86^Sr values of freshwater endmember (rivers) entering the bay.

The variability of ^87^Sr/^86^Sr values of the San Francisco Estuary and the Sacramento-San Joaquin Delta has been characterized previously in several studies and shown to be useful to reconstruct low-salinity conditions (<6 PSU) with high accuracy, but at higher salinities (>6 PSU) the concentration of ocean Sr dominates the mixing process resulting in poorer salinity resolution ^17^. However, these studies utilized only a single freshwater endmember (Sacramento River) in their mixing models, derived from water samples collected by Ingram and Sloan ^18^ or assumed freshwater salinity to be 0 PSU ^19^. The Delta receives freshwater from multiple source rivers including the Sacramento River from the north, the Cosumnes River and Mokelumne River from the east and the San Joaquin River from the south, each having relatively unique Sr concentrations, salinities, and ^87^Sr/^86^Sr values, which could influence the mixing process and subsequent estimates of low-salinity values from water ^87^Sr/^86^Sr. The Sacramento River is the dominant source of freshwater to the estuary in most years, since much of the San Joaquin River flows are diverted in the South Delta before reaching the confluence of the Sacramento and San Joaquin Rivers. Flows from the tributaries entering the Delta are highly regulated, monitored and tracked using a variety of modelling approaches ^20,21^. Flows exiting the Delta (hereinafter Outflow) and mixing with bay and ocean water are estimated using a 1-D hydrodynamic model rather than measured flows due to the inherent challenges with accurately accounting for tidal flows and in-delta consumption and discharge from agriculture. The California Department of Water Resources maintains this database and data are available from (http://water.ca.gov/dayflow/). This model also includes flow estimates from tributaries entering the Delta and water diverted by the CVP and SWP (Exports).

## Mixing Model Formulations

The mixing dynamics of freshwater and seawater in the San Francisco Estuary have been previously studied by Ingram and Sloan as well as by Phillis et al ^16,18^. In these studies, the estimated salinity of a mixture assuming a simple two-endmember mixing model derived from Faure and Mensing ^12^ as used; however, in Phillis, et al. ^18^ a simplifying assumption of 0 PSU for freshwater was used to simplify algebraic derivation from the original mixing model. Here, we derived the equation for estimating salinity form the two-endmember mixing model without this assumption.

*Term definitions:*

R_A_ = Strontium ratio of endmember A

R_B_ = Strontium ratio of endmember B

R_m_ = Strontium ratio of the mixed water (for a given mix F)

C_A_ = Strontium concentration of endmember A

C_B_ = Strontium concentration of endmember B

C_M_ = Strontium concentration of the mixed water (for a given mix F)

S_A_ = Salinity of endmember A

S_B_ = Salinity of endmember B

S_M_ = Salinity of the mixed water (for a given mix F)

F = Proportion of endmember A in the mixture (from 0 to 1)

We used a simple two-endmember mixing model (Faure, 1986). The salinity of a mixture is the salinity of each endmember multiplied by its proportional contribution to the sample:

1. $S_{M}= S_{A}*F+ S_{B}(1-F)$

Where the proportion of endmember A can be estimated from:

1. $F= \frac{S_{M}-S_{B}}{S_{A}-S_{B}}$

Similarly, the strontium concentration mixing equation for two endmembers:

(3) $C_{M}= C_{A}*F+ C_{B}(1-F)$

Combining these two mixtures provides the simple two-endmember model in Faure (1986). Strontium ratio mixing equation:

(4) $R_{M}=R_{A}* C_{A}*\left( \frac{F}{C_{M}} \right)+ R_{B}* C_{B}*\left( \frac{1-F}{C_{M}} \right)$

Sub out C_M_:

(5) $R_{M}=R_{A}* C_{A}*\left( \frac{F}{C_{A}*F+ C_{B}(1-F)} \right)+ R_{B}* C_{B}*\left( \frac{1-F}{C_{A}*F+ C_{B}(1-F)} \right)$

Solve for F:

(6) $R_{M}=\left( \frac{R_{A} C_{A}F}{C_{A}*F+ C_{B}(1-F)} \right)+ \left( \frac{R_{B}C_{B}-R_{B}C_{B}F}{C_{A}*F+ C_{B}(1-F)} \right)$

$$R_{M}=\frac{R_{A} C_{A}F+R_{B}C_{B}-R_{B}C_{B}F}{C_{A}F+ C_{B}-C_{B}F}$$

$$R_{M}\left( C_{A}F+ C_{B}-C_{B}F \right)=R_{A} C_{A}F+R_{B}C_{B}-R_{B}C_{B}F$$

$$R_{M}C_{A}F+ {R_{M}C}_{B}-{R_{M}C}_{B}F= R_{A} C_{A}F+R_{B}C_{B}-R_{B}C_{B}F$$

$$R_{M}C_{A}F -{R_{M}C}_{B}F-R_{A} C_{A}F+R_{B}C_{B}F= R_{B}C_{B}-{R_{M}C}_{B}$$

$$F\left( R_{M}C_{A} -{R_{M}C}_{B}-R_{A} C_{A}+R_{B}C_{B} \right)= R_{B}C_{B}-{R_{M}C}_{B}$$

$$F= \frac{R_{B}C_{B}-{R_{M}C}_{B}}{R_{M}C_{A} -{R_{M}C}_{B}-R_{A} C_{A}+R_{B}C_{B}}$$

Substitute F for the salinity mixing equation:

(7) $\frac{S_{M}-S_{B}}{S_{A}-S_{B}}= \frac{R_{B}C_{B}-{R_{M}C}_{B}}{R_{M}C_{A} -{R_{M}C}_{B}-R_{A} C_{A}+R_{B}C_{B}}$

$$S_{M}-S_{B}=\frac{R_{B}C_{B}-{R_{M}C}_{B}}{R_{M}C_{A} -{R_{M}C}_{B}-R_{A} C_{A}+R_{B}C_{B}}*(S_{A}-S_{B})$$

$$S_{M}-S_{B}=\frac{{S_{A}R}_{B}C_{B}-{S_{A}R_{M}C}_{B}- {S_{B}R}_{B}C_{B}+S_{B}{R_{M}C}_{B}}{R_{M}C_{A} -{R_{M}C}_{B}-R_{A} C_{A}+R_{B}C_{B}}$$

Final equation for the salinity of a mixture:

(8) $S_{M}=\left( \frac{{S_{A}R}_{B}C_{B}-{S_{A}R_{M}C}_{B}- {S_{B}R}_{B}C_{B}+S_{B}{R_{M}C}_{B}}{R_{M}C_{A} -{R_{M}C}_{B}-R_{A} C_{A}+R_{B}C_{B}} \right)+S_{B}$

***Validation of the mixing model for 2011-2012***

Flow data from October 1, 2011 to May 31, 2012 are shown in Supplement Fig. S1. 2011 was a wet year in the Central Valley of California but transitioned into a dry year in 2012. Sacramento River flows comprised the bulk of the inflows (76%) to the Delta and Delta Outflow (92%) during this time period except for a brief flow pulse from the Yolo Bypass, a large man-made floodplain engineered to protect the city of Sacramento from flooding. The rivers flowing into the Delta from the east, Cosumnes and Mokelumne River comprise a very small proportion (~3%) of flows into the Delta. In 2011, the San Joaquin River was the second largest contributor to flows to the Delta comprising ~30% of flows into the Delta from January to August but only 12% during this period in 2012 when exports at the time exceeded San Joaquin River flows. These data would suggest that using the Sacramento River freshwater endmember would provide a reasonable approximation to the mixing of freshwater and ocean ^87^Sr/^86^Sr values. However, since Delta Smelt can spawn in the south Delta and potentially in the lower reaches of the San Joaquin River, we included the San Joaquin River in our mixing model (Supplement Fig. S2).

To parameterize the ^87^Sr/^86^Sr to salinity mixing model we used three water samples as endmembers. The marine endmember is based on sample collected in the Pacific Ocean at Muir Beach just north of the entrance to San Francisco Bay on 4/23/2007 with a Sr concentration of 7900 ppm and salinity of 31.8 PSU and an ^87^Sr/^86^Sr value of 0.70918. The Sacramento endmember was collected on the Lower Sacramento River (station 711) on 3/8/2018 with a Sr concentration of 95 ppm and a salinity of 0.11 and ^87^Sr/^86^Sr value of 0.70576. The San Joaquin endmember was collected at Mossdale (above station 912) on 4/25/2007 with a Sr concentration of 209 ppm and a salinity of 0.20 and ^87^Sr/^86^Sr value of 0.70714.

Theoretical mixing curves using only Sacramento River or only San Joaquin River water demonstrated the range of ^87^Sr/^86^Sr values for water with salinity values less than 0.5 PSU (Supplement Fig. S2). We then calculated a mixture of the freshwater endmembers as 70:30 Sacramento River and San Joaquin River which resulted in Sr concentration of 129 ppm, a salinity of 0.14 and ^87^Sr/^86^Sr value of 0.70643. This 70-30 mixture of the two rivers was used as the freshwater endmember in the salinity calculations.

We found that ^87^Sr/^86^Sr values can provide fine scale resolution for salinity below 6 PSU, but due to differences in Sr concentrations between fresh and ocean waters and resultant strong non-linear relationship between salinity and ^87^Sr/^86^Sr (Supplement Fig. S2), estimates of salinities above 6 PSU were less reliable. For this reason, any calculated salinity values in the life history chronologies above 6 PSU were truncated to 6 and any values of 6 were considered to be minimum salinity estimates. To validate our mixing model, water samples were collected throughout the San Francisco Estuary (Supplement Table S1), including samples from minor San Francisco Tributaries such as the Napa River. The salinity for the majority of water samples followed the strontium to salinity mixing line (Mean Absolute Error = 0.13 PSU). Additionally, the model explained ~98% of the variance in the water sample salinity.

Figure S1: A. Daily mean flows (cubic feet per second-CFS) into the Delta from Oct 1, 2011 to Jun 1, 2012. B. Sacramento River flows entering the Delta, Export volume pumped out of the Delta and flows exiting the Delta into the bay.


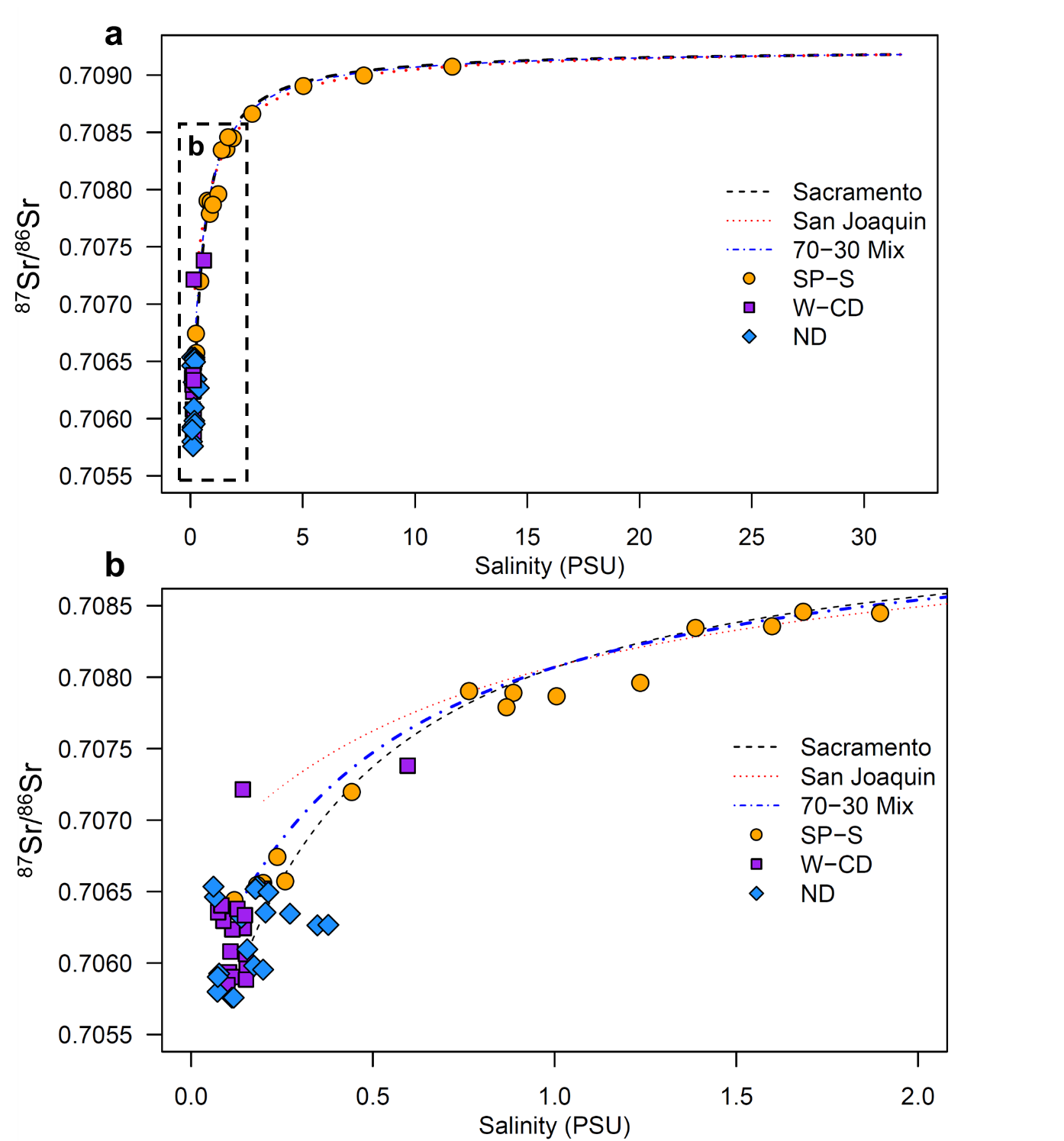


Figure S2: Strontium isotope mixing relationship between the Sacramento River (dashed black line), San Joaquin River (dot red line) and the Pacific Ocean. Based on the average proportional flows from the Sacramento and San Joaquin contributing to the estuary, the dashed line depicts the mixing dynamics in 2012 (70-30 Mix, dot-dash blue). Individual water samples are demarked by shapes corresponding to collection locations. **a** is the full mixing model while **b** focuses on the low salinity portion of the curve between 0 and 2 PSU.

## Jackknife classification results

Table S11: Jackknife classification results for the four semi-anadromous clusters using natal chemistry, chemistry post dispersal, and age at transition with an overall classification success rate of 90%.

|  | Classified | | | |
| --- | --- | --- | --- | --- |
| Actual | Cluster3 | Cluster4 | Cluster5 | Cluster6 |
| Cluster3 | 13 (93%) | 1 (7%) | 0 (0%) | 0 (0%) |
| Cluster4 | 2 (3%) | 65 (88%) | 6 (8%) | 1 (1%) |
| Cluster5 | 0 (0%) | 2 (2%) | 91 (96%) | 2 (2%) |
| Cluster6 | 0 (0%) | 0 (0%) | 8 (17%) | 40 (83%) |

Table S12: Jackknife classification results for all 6 clusters using natal chemistry and chemistry post dispersal with an overall classification success rate of 69%. The overall low classification rate was driven by the inability to differentiate the semi-anadromous clusters (5 and 6) without using age at transition. Classification between cluster 1 and 2 was high 97% and 95%, respectively.

|  | Classified | | | | | |
| --- | --- | --- | --- | --- | --- | --- |
| Actual | Cluster1 | Cluster2 | Cluster3 | Cluster4 | Cluster5 | Cluster6 |
| Cluster1 | 33 (97%) | 0 (0%) | 1 (3%) | 0 (0%) | 0 (0%) | 0 (0%) |
| Cluster2 | 0 (0%) | 18 (95%) | 0 (0%) | 0 (0%) | 0 (0%) | 1 (5%) |
| Cluster3 | 0 (0%) | 0 (0%) | 11 (73%) | 4 (27%) | 0 (0%) | 0 (0%) |
| Cluster4 | 0 (0%) | 0 (0%) | 3 (4%) | 55 (74%) | 14 (19%) | 2 (3%) |
| Cluster5 | 0 (0%) | 0 (0%) | 0 (0%) | 14 (15%) | 80 (84%) | 1 (1%) |
| Cluster6 | 0 (0%) | 1 (2%) | 0 (0%) | 8 (17%) | 39 (81%) | 0 (0%) |

Table S13: Jackknife classification results for all 6 clusters with the semi-anadromous clusters (3-6) combined into one group, using natal chemistry and chemistry post dispersal with an overall classification success rate of 99%. Classification between cluster 1 and 2 was high 97% and 95%, respectively.

|  | Classified | | |
| --- | --- | --- | --- |
| Actual | Cluster1 | Cluster2 | Cluster3-6 |
| Cluster1 | 33 (97%) | 0 (0%) | 1 (3%) |
| Cluster2 | 0 (0%) | 18 (95%) | 1 (5%) |
| Cluster3-6 | 0 (0%) | 1 (0.4%) | 231 (99%) |

## Capture region proportions for life history types.

Table S14: The number and percentage of life history types captured in the regions of the SFE. FWR = freshwater resident-cluster 1, BWR = brackish water resident-cluster 2, SA – semi-anadromous clusters 3-6. SP-S = San Pablo-Suisun Bay, W-CD = West-Central Delta, ND = North Delta. n represents the number of fish assigned to life history type from the hierarchical clustering and the percentage refers to the proportion of life history types caught in different regions. Note that during Jan-May the ND was always freshwater, while the boundary for freshwater and brackish water was located within the W-CD and the SP-S region was brackish.

|  | | SP-S | | | W-CD | | | ND | |
| --- | --- | --- | --- | --- | --- | --- | --- | --- | --- |
|  | n | | % | n | | % | n | | % |
| FWR | 0 | | 0% | 0 | | 0% | 29 | | 100% |
| BWR | 5 | | 45% | 2 | | 18% | 4 | | 36% |
| SA | 55 | | 45% | 25 | | 20% | 43 | | 35% |

## Cluster reproduction proportions

We used the mature individuals in the clustered data set (those caught in the SKT survey Jan-May) to examine the relationship between life history types and reproduction. We aggregated reproductive stages into early (Stages I – III) and late (IV – VI) as defined by CDFW.

Table S15: Number and proportion of fish in each cluster that were non reproductive or reproductive in the San Pablo/Suisun Bay region. Non-reproductive were fish in CDFW gonad stages I-III and reproductive were fish in CDFW gonad stages IV-VI.

|  |  | SP-S | | | | W-CD | | | | ND | | | | | |
| --- | --- | --- | --- | --- | --- | --- | --- | --- | --- | --- | --- | --- | --- | --- | --- |
|  |  | Early-Stage Gonads | | Late-Stage Gonads | | Early-Stage Gonads | | Late-Stage Gonads | | Early-Stage Gonads | | Late-Stage Gonads | |  |  |
|  |  | M | F | M | F | M | F | M | F | M | F | M | F | |  |
|  | FWR-Cluster1 | 0 | 0 | 0 | 0 | 0 | 0 | 0 | 0 | 5 | 14 | 5 | 5 | |  |
|  | BWR-Cluster2 | 0 | 3 | 1 | 0 | 0 | 1 | 0 | 1 | 0 | 1 | 1 | 2 | |  |
|  | SA-Cluster3 | **0** | 0 | 0 | 0 | 0 | 1 | 0 | 0 | 0 | 2 | 2 | 0 | |  |
|  | SA-Cluster4 | 2 | 4 | 0 | 1 | 4 | 2 | 1 | 0 | 10 | 10 | 2 | 1 | |  |
|  | SA-Cluster5 | 11 | 18 | 1 | 1 | 1 | 6 | 3 | 1 | 1 | 4 | 3 | 2 | |  |
|  | SA-Cluster6 | 7 | 5 | 0 | 5 | 2 | 2 | 2 | 2 | 0 | 1 | 1 | 4 | |  |
|  |  |  |  |  |  |  |  |  |  |  |  |  |  | |  |
|  | SA Totals by stage and region | 20 | 27 | 1 | 7 | 7 | 11 | 6 | 3 | 11 | 17 | 8 | 7 | |  |
|  |  |  |  |  |  |  |  |  |  |  |  |  |  | |  |
|  | SA % mature by region |  |  |  | 15% |  |  |  | 33% |  |  |  | 35% | |  |
|  | SA % female mature by region |  |  |  | 21% |  |  |  | 21% |  |  |  | 29% | |  |
|  | SA % males mature by region |  |  | 5% |  |  |  | 46% |  |  |  | 42% |  | |  |

## Reproduction Logistic Regressions

Table S16: Model comparison table detailing which terms were included and the adjusted R^2^, degrees of freedom, log likelihood, and AICc of each model, sorted by increasing AICc. The full model (first in the table) was selected.

| (Int) | Reg | JD | Sex | Reg:JD | Reg:Sex | JD:Sex | Reg:JD:Sex | adjR^2 | df | logLik | AICc | delta | weight |
| --- | --- | --- | --- | --- | --- | --- | --- | --- | --- | --- | --- | --- | --- |
| -7.904 | + | 0.08015 | + | + | + | + | + | 0.5779 | 12 | -329.41 | 683.1 | 0 | 0.997 |
| -6.04 | + | 0.06 | + | + | + | + |  | 0.5641 | 10 | -337.28 | 694.8 | 11.63 | 0.003 |
| -5.217 | + | 0.05068 | + | + | + |  |  | 0.5557 | 9 | -342.06 | 702.3 | 19.16 | 0 |
| -6.723 | + | 0.0675 | + |  | + | + |  | 0.551 | 8 | -344.67 | 705.5 | 22.34 | 0 |
| -5.821 | + | 0.05755 | + |  | + |  |  | 0.5429 | 7 | -349.21 | 712.5 | 29.39 | 0 |
| -8.385 | + | 0.07874 | + | + |  | + |  | 0.5202 | 8 | -361.61 | 739.4 | 56.23 | 0 |
| -7.663 | + | 0.06979 | + |  |  | + |  | 0.5156 | 6 | -364.07 | 740.2 | 57.09 | 0 |
| -6.925 | + | 0.06241 | + | + |  |  |  | 0.5015 | 7 | -371.6 | 757.3 | 74.18 | 0 |
| -6.213 | + | 0.05367 | + |  |  |  |  | 0.4973 | 5 | -373.86 | 757.8 | 74.64 | 0 |
| -6.632 |  | 0.06672 | + |  |  | + |  | 0.4855 | 4 | -380.01 | 768.1 | 84.93 | 0 |
| -5.384 |  | 0.05282 | + |  |  |  |  | 0.4688 | 3 | -388.64 | 783.3 | 100.18 | 0 |
| -5.282 | + | 0.05099 |  | + |  |  |  | 0.4152 | 6 | -415.46 | 843 | 159.86 | 0 |
| -4.55 | + | 0.04207 |  |  |  |  |  | 0.4106 | 4 | -417.7 | 843.4 | 160.3 | 0 |
| -3.86 |  | 0.04164 |  |  |  |  |  | 0.3826 | 2 | -431.06 | 866.1 | 183 | 0 |
| -1.534 | + |  | + | + |  |  |  | 0.1015 | 6 | -549.09 | 1110.3 | 427.13 | 0 |
| -1.942 | + |  | + |  |  |  |  | 0.0745 | 4 | -559.12 | 1126.3 | 443.14 | 0 |
| -1.748 | + |  |  |  |  |  |  | 0.06117 | 3 | -564 | 1134 | 450.89 | 0 |
| -1.227 |  |  | + |  |  |  |  | 0.01287 | 2 | -581.3 | 1166.6 | 483.47 | 0 |
| -1.054 |  |  |  |  |  |  |  | 0 | 1 | -585.81 | 1173.6 | 490.49 | 0 |

Table S17: Analysis of Deviance table comparing the full model to the null model, using the ANOVA function and χ^2^ test in R.

| Model 1: Stage ~ JD * Sex * Reg | | | | |  | |  |
| --- | --- | --- | --- | --- | --- | --- | --- |
| Model 2: Stage ~ 1 (Null model) | | | | |  | |  |
|  | Resid. Df | Resid. Dev | Df | Deviance | | Pr(>Chi) | |
| 1 | 1013 | 658.82 |  |  | |  | |
| 2 | 1024 | 1171.62 | -11 | -512.79 | | <2.2e-16 | |

Table S18 Analysis of Deviance table comparing the full model to the next most explanatory model using the ANOVA function and χ^2^ test in R.

| Model 1: Stage ~ JD * Sex * Reg | | | | |  |
| --- | --- | --- | --- | --- | --- |
| Model 2: Stage ~ JD + Reg + Sex + Reg:JD + JD:Sex | | | | | |
|  | Resid. Df | Resid. Dev | Df | Deviance | Pr(>Chi) |
| 1 | 1013 | 658.82 |  |  |  |
| 2 | 1017 | 723.22 | -4 | -64.398 | 3.45E-13 |

***Supplementary References***

1 Nichols, F. H., Cloern, J. E., Luoma, S. N. & Peterson, D. H. The modification of an estuary. *Science* **231**, 567-573 (1986).

2 Winder, M. & Jassby, A. D. Shifts in zooplankton community structure: implications for food web processes in the upper San Francisco Estuary. *Estuaries and Coasts* **34**, 675-690 (2011).

3 Cloern, J. E. & Jassby, A. D. Drivers of change in estuarine-coastal ecosystems: Discoveries from four decades of study in San Francisco Bay. *Reviews of Geophysics* **50**, doi:10.1029/2012rg000397 (2012).

4 SWRCB. in *Water Rights Decision 1641* Vol. D-1641 (ed State of California State Water Resources Control Board) (Sacramento Ca. , 1999).

5 Hobbs, J. A., Bennett, W. A., Burton, J. E. & Baskerville-Bridges, B. Modification of the biological intercept model to account for ontogenetic effects in laboratory-reared delta smelt (Hypomesus transpacificus). *Fish B-Noaa* **105**, 30-38 (2007).

6 Willmes, M., Glessner, J. J. G., Carleton, S. A., Gerrity, P. C. & Hobbs, J. A. 87Sr/86Sr isotope ratio analysis by laser ablation MC-ICP-MS in scales, spines, and fin rays as a non-lethal alternative to otoliths for reconstructing fish life history. *Canadian Journal of Fisheries and Aquatic Sciences*, doi:10.1139/cjfas-2016-0103 (2016).

7 McArthur, J. M., Howarth, R. & Bailey, T. Strontium isotope stratigraphy: LOWESS version 3: best fit to the marine Sr-isotope curve for 0–509 Ma and accompanying look-up table for deriving numerical age. *The Journal of Geology* **109**, 155-170 (2001).

8 Mokadem, F. *et al.* High-precision radiogenic strontium isotope measurements of the modern and glacial ocean: Limits on glacial-interglacial variations in continental weathering. *Earth Planet Sc Lett* **415**, 111-120, doi:10.1016/j.epsl.2015.01.036 (2015).

9 Walther, B. D. & Limburg, K. E. The use of otolith chemistry to characterize diadromous migrations. *Journal of Fish Biology* **81**, 796-825, doi:10.1111/j.1095-8649.2012.03371.x (2012).

10 Bataille, C. P. & Bowen, G. J. Mapping Sr-87/Sr-86 variations in bedrock and water for large scale provenance studies. *Chem Geol* **304**, 39-52, doi:10.1016/j.chemgeo.2012.01.028 (2012).

11 Bataille, C. P. *et al.* A geostatistical framework for predicting variations in strontium concentrations and isotope ratios in Alaskan rivers. *Chem Geol* **389**, 1-15, doi:10.1016/j.chemgeo.2014.08.030 (2014).

12 Hodell, D. A. *et al.* Variations in the strontium isotope composition of seawater during the Paleocene and early Eocene from ODP Leg 208 (Walvis Ridge). *Geochem Geophy Geosy* **8**, doi:Artn Q09001 Doi 10.1029/2007gc001607 (2007).

13 Faure, G. & Mensing, T. M. Isotopes. *Principles and applications* **897** (2005).

14 Ingram, B. L. & DePaolo, D. J. A 4300 year strontium isotope record of estuarine paleosalinity in San Francisco Bay, California. *Earth Planet Sc Lett* **119**, 103-119 (1993).

15 Shao, Y. *et al.* Calcium and strontium isotope systematics in the lagoon-estuarine environments of South Australia: Implications for water source mixing, carbonate fluxes and fish migration. *Geochim Cosmochim Ac* **239**, 90-108 (2018).

16 Walther, B. D. & Nims, M. K. Spatiotemporal Variation of Trace Elements and Stable Isotopes in Subtropical Estuaries: I. Freshwater Endmembers and Mixing Curves. *Estuaries and Coasts* **38**, 754-768, doi:10.1007/s12237-014-9881-7 (2015).

17 Hobbs, J. A., Lewis, L. S., Ikemiyagi, N., Sommer, T. & Baxter, R. D. The use of otolith strontium isotopes (Sr-87/Sr-86) to identify nursery habitat for a threatened estuarine fish. *Environmental Biology of Fishes* **89**, 557-569, doi:10.1007/s10641-010-9672-3 (2010).

18 Ingram, B. & Sloan, D. Strontium isotopic composition of estuarine sediments as paleosalinity-paleoclimate indicator. *Science* **255**, 68-72 (1992).

19 Phillis, C. C., Ostrach, D. J., Ingram, B. L. & Weber, P. K. Evaluating otolith Sr/Ca as a tool for reconstructing estuarine habitat use. *Canadian Journal of Fisheries and Aquatic Sciences* **68**, 360-373, doi:doi:10.1139/F10-152 (2011).

20 MacWilliams, M. L., Ateljevich, E. S., Monismith, S. G. & Enright, C. An Overview of Multi-Dimensional Models of the Sacramento–San Joaquin Delta. *San Francisco Estuary and Watershed Science* **14** (2016).

21 MacWilliams, M. L., Bever, A. J., Gross, E. S., Ketefian, G. S. & Kimmerer, W. J. Three-Dimensional Modeling of Hydrodynamics and Salinity in the San Francisco Estuary: An Evaluation of Model Accuracy, X2, and the Low–Salinity Zone. *San Francisco Estuary and Watershed Science* **13** (2015).
